# T Cell Dysregulation in Non-silicotic Silica Exposed Workers: A Step Toward Immune Tolerance Breakdown

**DOI:** 10.3389/fimmu.2019.02743

**Published:** 2019-11-22

**Authors:** Benoit Brilland, Céline Beauvillain, Gery Mazurkiewicz, Pierre Rucay, Yves Roquelaure, Julie Tabiasco, Emeline Vinatier, Jérémie Riou, Pascale Jeannin, Gilles Renier, Jean-François Subra, Jean-François Augusto

**Affiliations:** ^1^Service de Néphrologie-Dialyse-Transplantation, CHU d'Angers, Angers, France; ^2^CRCINA, INSERM, Université de Nantes, Université d'Angers, Angers, France; ^3^Laboratoire d'Immunologie et d'Allergologie, CHU d'Angers, Angers, France; ^4^Service Santé au Travail Côte de Lumière, Les Sables-d'Olonne, France; ^5^Service de Médecine du Travail, CHU d'Angers, Angers, France; ^6^MINT, UNIV Angers, INSERM 1066, CNRS 6021, IBS- CHU, Angers, France

**Keywords:** crystalline silica, occupational diseases, auto-immune diseases, activated T cells, regulatory T cells, auto-antibodies

## Abstract

**Background:** Chronic silica exposure can lead to silicosis, complicated or not by autoimmune diseases (AID). The pathophysiology of silica-induced AID remains not fully understood, especially immune mechanisms that may develop in patients without yet established silicosis. We conducted a prospective clinical study to analyze the impact of crystalline silica (CS) on T cell phenotype and regulatory T cells (Tregs) frequency, as well as on auto-antibodies development in non-silicotic workers exposed to CS.

**Methods:** Workers with moderate to high exposure level to CS and aged between 30 and 60 years-old were considered for inclusion. Peripheral blood mononuclear cells were analyzed by flow cytometry. Auto-antibodies were screened in serum by immunofluorescence. Blood from 42 and 45 healthy subjects (HC) was used as control for T cell phenotype and serum analyses, respectively.

**Results:** Among the 63 included workers exposed to CS, 55 had full data available and were analyzed. Ten were exposed to CS for <5 years, 18 for 5–10 years and 27 for more than 10 years. The frequency of Tregs (CD4^+^CD25^+^CD127^−^FoxP3^+^) was significantly lower in CS exposed workers as compared to HC. We found an increased expression of the activation marker HLA-DR on T cells (CD3^+^, CD4^+^, and CD8^+^) of CS exposed workers as compared to HC. Tregs to activated T cells ratio was also lower in exposed subjects. In the latter, HLA-DR expression level and Tregs frequency were significantly associated with CS exposure duration. Serum autoantibody detection was significantly higher in CS exposed workers as compared to HC. Especially, among workers exposed more than 10 years, antinuclear antibodies and ANCA were detected in 44 and 22% among them, as compared to 5 and 2.5% in HC, respectively.

**Conclusion:** This work shows that CS exposure is associated with a decrease of Tregs frequency, an increase of T cell activation status, and a tolerance breakdown against auto-antigens. These results show that alterations of the T cell compartment can be detected early over the course of CS exposure, preceding silicosis development or AID onset.

## Introduction

Crystalline silica (CS) dust exposure is a common feature of sand or rock mining. Construction and ceramics production workers are also largely exposed to CS. Indeed, silica is a major component of Earth composition (1). Recently, workers in fashion industry (sand blasting) have also been recognized as highly exposed to CS. A major burden of silica exposure is silicosis (2), which is, with asbestosis, among the most frequent pneumoconiosis. This occupational lung disease presents as a progressive lung fibrosis characterized by parenchymal inflammation and nodular lesions. The pathophysiology of silicosis has been largely studied. Schematically, after phagocytosis of inhaled silica by alveolar macrophages, inflammasome activation leads to pro-inflammatory cytokines release. Macrophages, which are unable to destruct silica, undergo apoptosis; silica is then released and triggers another cycle of phagocytosis and inflammation. This chronic inflammation leads to excessive collagen production by fibroblast, leading to fibrosis (2).

Since the beginning of the last century, increased prevalence of autoimmune diseases (AID) has been observed in subjects exposed to silica, whether they suffer from silicosis or not. Rheumatoid arthritis (RA) (3, 4), systemic sclerosis (SSc) (5), systemic lupus erythematosus (SLE) (6–9) and ANCA-associated vasculitis (AAV) (10–12) represent the main AID which are over-represented in silica-exposed workers. The pathophysiology of these diseases in the context of silica exposure is far from being well understood and relies largely on data acquired in silicotic patients.

Several studies reported the presence of peripheral lymphopenia in silicotic patients with or without AID (13, 14). We hypothesized in the present study that the lymphopenia may impact preferentially regulatory T cells (Tregs) and could account for auto-immunity development. Thus, as observed in other diseases, decreased Tregs could be followed by a break of tolerance against self-antigens and could be responsible for the occurrence of AID.

To better understand early immune mechanisms, we studied a cohort of 63 silicosis-free subjects, exposed to CS, age- and sex- matched with healthy donors (HC). We analyzed the phenotype of their leucocyte by flow cytometry and screened serum markers of autoimmunity. We hereby demonstrate that, compared to HC, CS exposed workers have decreased Tregs frequency and increased T cell activation status. Moreover, CS exposed workers showed a higher frequency of ANA and ANCA positivity, reflecting B cell activation.

## Materials and Methods

### Study Design, Patients, and Sample Collection

This prospective study was conducted from January 2008 to July 2009. Silica-exposed workers were enrolled according to the following inclusion criteria: male aged between 30 and 60 years-old without history of pulmonary or rheumatic disease, exposed to CS in one of the companies included in the study, as described below. Exclusion criteria were age below 30 or above 60, female gender, past medical history of lung disease (tuberculosis, COPD, lung cancer), infectious disease or immunosuppressive regimen at the time of the study inclusion, auto-immune disease, radiologic abnormality suggestive of silicosis, asbestosis, anthracosis, or tuberculosis. Blood from age- and sex-matched HC was obtained from human volunteers (Blood collection center, Angers, France; Agreement PLER ANG 2017-01).

Companies with a common medical supervision and with available metrological data on the exposure of employees to free silica were selected. For this, the COLCHIC database from the “Institut National de Recherche et Sécurité” (15, 16) and files from the “Caisse d'Assurance Retraite et de la Santé au Travail” and the “Laboratoire Interrégional de Chimie de l'Ouest” were used. Free-silica exposure was ranked as “low,” “moderate” or “high” in respect to CS exposure and according to the metrological data from the COLCHIC database: sampling used an air sampler that captures the respirable (alveolar) fraction of airborne particles (European Standard CN 481). Average exposures above 70% of the mean exposure value (MEV), or above the MEV are considered “high,” exposures between 30 and 70% of the MEV are considered “moderate” and those <30% of the MEV are considered “low” (17). Workers with moderate to high exposure were eligible for inclusion in the present study. After information about the study, randomly drawn exposed subjects who agreed to participate underwent clinical examination, chest X-ray, pulmonary function test and blood sampling.

This study followed the recommendations of the Helsinki Declaration and was approved by the ethical committee of the Angers University Hospital (CCPPRB 2006/23 and 2006/23 bis).

### Autoantibody Detection

Autoantibodies were detected in the serums of workers exposed to SC and in HC. ANA were detected by indirect immunofluorescence (IIF) on HEp2 cells (Bio-Rad) (18). Given the age of workers exposed to CS, ANA were considered positives when the dilution titer was ≥1/200th. ANCA were detected by IIF on ethanol fixed normal human neutrophils, starting the screening at a dilution of 1/20th, and were classified as cytoplasmic (cANCA), perinuclear (pANCA) or atypical (aANCA) (19). In case of IIF positivity, antimyeloperoxidase (MPO) and antiproteinase 3 (PR3) antibodies were detected using ELISA (all from Euroimmun). Rheumatoid factors (RF) were measured in serum by nephelometry and anti-citrullinated protein antibodies (ACPA) were detected by fluoroenzymology (Bio-Rad). Complete blood count was obtained from total blood on a routine automated hematology analyzer.

### Flow Cytometry Analysis

**Figure 1:**
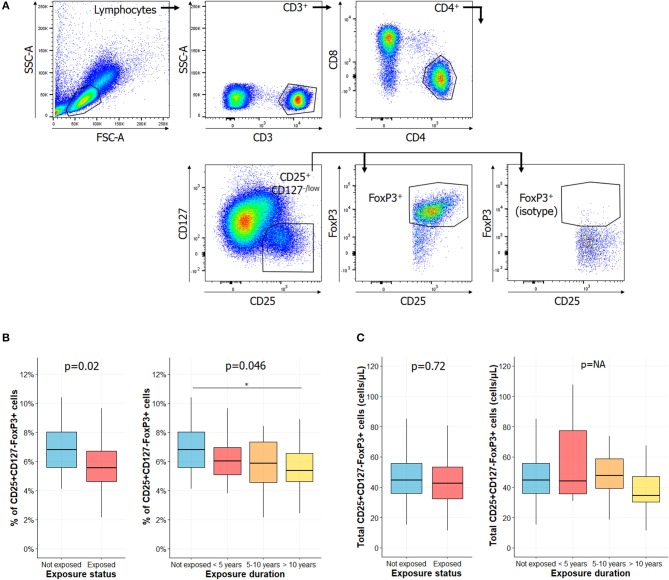
Percentage of regulatory T cell is lower in exposed subjects and decreased with longer CS exposure. **(A)** Gating strategies for regulatory T cells (Tregs), as described in Santegoets et al. (20): lymphocytes were identified based on their morphology characteristics, as shown in the forward scatter (FSC) and side scatter (SSC) plot. Among these cells, the following populations were successively gated in to identify Tregs: CD3^+^ cells, CD4^+^ cells, CD25^+^CD127^−/low^, and FoxP3^+^ cells. Percentage of Tregs among CD4^+^ cells of subjects exposed or not to silica (**B**, left) or in subgroups of subjects exposed to silica (**B**, right). Total counts of circulating Tregs in subjects exposed or not to silica (**C**, left) or in subgroups of subjects exposed to CS (**C**, right). Data are shown as boxplot. *P*-values represent Mann & Whitney test for two groups comparison and, Kruskal-Wallis test for four groups comparison (if previous test significant), followed by *post-hoc* Dunn test when applicable (results indicated by asterisks, *). *P*-values have been adjusted according to Hochberg method to account for repeated analysis. NA, not applicable.

**Figure 2:**
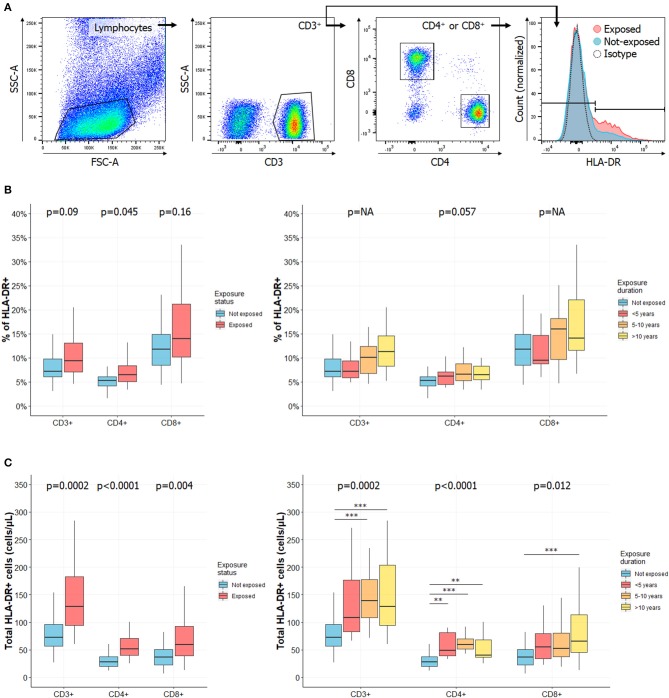
Frequency of activated T cells is increased in CS exposed workers as compared to HC and increases with exposure length. **(A)** Gating strategies for activated T cells. Lymphocytes were identified based on their morphology characteristics [forward scatter (FSC) and side scatter (SSC) plot]. Among these cells, total CD3^+^ T cells were identified as well as CD4^+^ and CD8+ subsets. Activated cells were defined according to HLA-DR expression. Percentages of HLA-DR^+^ in CD3^+^, CD4^+^, or CD8^+^ cells in subjects exposed or not to CS (**B**, left) or in subgroups of subjects exposed to CS (**B**, right). Total counts of activated HLA-DR^+^ CD3^+^, CD4^+^, or CD8^+^ T cells in subjects exposed or not to silica (**C**, left) or in subgroups of subjects exposed to silica (**C**, right). Data are shown as boxplot. *P*-values represent Mann & Whitney test for two groups comparison and, Kruskal-Wallis test for four groups comparison (if previous test significant), followed by *post-hoc* Dunn test when applicable (results indicated by asterisks, *). *P*-values have been adjusted according to Hochberg method to account for repeated analysis. NA, not applicable.

Peripheral blood mononuclear cells (PBMC) were isolated from CS exposed workers and HC using standard density-gradient centrifugation on Lymphocyte Separation Medium (Eurobio). 10^6^ cells were incubated with antibodies listed in Supplemental Table 1 (30 min, 4°C). For Tregs, after fixation and permeabilization (Intracellular Fixation & Permeabilization Buffer Set, eBioscience), cells were incubated for 30 min with anti-FoxP3 antibody. As commonly accepted, Tregs were defined as CD4^+^CD25^+^CD127^−/low^FoxP3^+^ cells (Figure 1A) (20). For T cell activation assessment, 100 μL of total blood from EDTA tube was incubated (15 min, room temperature) with indicated antibodies (Supplemental Table 1). Red blood cells were lysed before flow cytometry analysis. Activated T cells were defined as CD3^+^HLA-DR^+^, CD3^+^CD4^+^HLA-DR^+^ and CD3^+^CD8^+^HLA-DR^+^ cells (Figure 2A) (21, 22).

Isotype antibodies or unstained cells were used as negative controls for gating strategies. Cells were immediately read with cytometer (FACSCanto II, BD Bioscience) and spectral overlap was compensated within DIVA software (BD Bioscience) before acquisition. Events were analyzed with FlowJo v10.3 software (BD Bioscience).

### Statistical Analyses

Subjects' characteristics are reported as numbers and percentages for qualitative variables, and with mean ± standard deviation, or median [Inter-Quartile Range (IQR)], when appropriated, for continuous variables. Data were compared using the Fisher exact test for categorical variables or Student *t*-test or ANOVA (followed by Tukey *post-hoc* test for multiples comparisons) for normal continuous variables. For non-normal continuous variables, Mann-Whitney or Kruskal-Wallis tests (followed by Dunn *post-hoc* test for multiples comparisons when applicable) were used. For multiple comparisons, *P*-values were adjusted using the Hochberg procedure, which allows a control of the Family-wise Error Rate at 5% (23, 24). For flow cytometry analysis and total blood count analysis, Mann Whitney U test was followed by Kruskal-Wallis test only if *p*-value was significant. *P*-values have been adjusted according to Hochberg method to account for repeated analysis.

Data are shown as boxplot (median ± interquartile range). Vertical bar represents minimum and maximum values. Statistical analysis was performed using the R software version 3.6. *P*-values <0.05 were considered significant (^*^ = *p* < 0.05, ^**^ = *p* < 0.01, ^***^ = *p* < 0.001).

## Results

### Population Characteristics

Complete data were available for 58 of 63 subject exposed to CS. Three additional subjects were excluded because of unexplained hyperleukocytosis (*n* = 1), hyperlymphocytosis (*n* = 1) or recent silicosis diagnosis (*n* = 1). Thus, 55 subjects were analyzed and were distributed as follows: subjects exposed to CS for <5 years (*n* = 10), for 5–10 years (*n* = 18), and for more than 10 years (*n* = 27). They were compared to age- and sex-matched healthy control (HC). Due to limitations in the total amount of drawable blood, two cohort of HC were constituted: *n* = 42 for hematological and flow cytometry analysis (HC group 1) and *n* = 45 for autoantibody analysis (HC group 2). The flow chart of the study is reported in Supplemental Figure 1.

All included subjects were males and worked in construction companies (daily activities reported in Supplemental Table 2). There was no difference in age between the exposed group or subgroups and HC group 1 or HC group 2 (Table 1). Most of exposed workers wore personal protective equipments (96.4%). There was a high proportion of smokers in each exposed subgroup (58.2% overall). Smokers were more common in the subgroup of subjects exposed 5–10 years (14/18; 78%). Alcohol consumption was significantly increased in subjects exposed more than 10 years (8/27; 29.7%) (Table 1).

**Table 1:** Demographical data.

	**Non-exposed control group**	**All exposed subjects** **(*n* = 55)**	**Exposed** **<5 years** **(*n* = 10)**	**Exposed** **5–10 years** **(*n* = 18)**	**Exposed** **>10 years** **(*n* = 27)**	***p*-values**
Sex ratio (♂/♀)	1/0	1/0	1/0	1/0	1/0	–
Age (years)	Control group 1: (*n* = 42) 41.67 ± 12.59 Control group 2: (*n* = 45) 42.06 ± 8.89	41.30 ± 6.52	38.23 ± 6.43	38.87 ± 6.18	44.07 ± 5.91	NS^‡^
PPE	–	53 (96.4%)	10 (100%)	18 (100%)	25 (92.6%)	0.67
BMI (kg/m^2^)	–	26.10 ± 4.56	24.55 ± 3.52	26.45 ± 5.38	26.44 ± 4.22	0.49
Smokers Active Past	– – –	32 (58.2%) 22 (68.6%) 11 (34.4%)	3 (30%) 3 (100%) 0 (0%)	14 (78%) 8 (57%) 6 (43%)	15 (55.6%) 11 (73.3%) 4 (26.7%)	**0.05** 0.87 0.08
Alcohol consumption	–	9 (16.4%)	0 (0%)	1 (5.6%)	8 (29.7%)	**0.05**
Current medication						
Occasional NSAID Anti-hypertensive Anti-diabetic Hypolipemic Bronchodilator	– – – – –	2 (3.6%) 4 (7.3%) 1 (1.8%) 2 (3.6%) 2 (3.6%)	1 (10%) – – – 1 (10%)	– 1 (5.6%) 1 (5.6%) 1 (5.6%) 1 (5.6%)	1 (3.7%) 3 (11.1%) – 1 (3.7%) –	0.43 0.66 0.51 1.00 0.24
Abnormal chest radiography	–	2 (7.4%)	0 (0%)	0 (0%)	2 (7.4%)^$^	0.70
Abnormal PFT	–	5 (9.1%)	0 (0%)	1 (5.8%)^€^	4 (14.8%)^€€^	0.58

*The bold value indicates that ≤ 0.05*.

*p-values are from Fisher exact test otherwise specified*.

‡ *Exposed subjects vs. control group 1 (flow cytometry analysis): p-value = 0.85 (T-test) and 0.22 (ANOVA). Exposed subjects vs. control group 2 (immunological analysis): p-value = 0.63 (T-test) and 0.07 (ANOVA)*.

$ *One subject with right subaxillary pleural thickening and one patient with increased peribronchial interstitium without interstitial syndrome*.

€ *One restrictive syndrome*.

€€ *Three obstructive syndromes, one NA*.

*CS, crystalline silica; PPE, personal protective equipment; BMI, body mass index; NS, not significant; NSAID, non-steroidal anti-inflammatory drugs; PFT, pulmonary function test*.

### Hematological Data

Complete blood count results are detailed in Supplemental Table 3. We found a slight but significant increase of hemoglobin level in subject exposed to CS, especially in the two subgroups with longer CS exposure, as compared to HC. There was a significant increase in total leucocytes and in total lymphocytes counts in subjects exposed to CS, especially in the subgroup exposed 5–10 years. We also noted a significant increase in total monocyte count in each subgroup compared to HC. Nevertheless, all these values remained within the normal range. CD3^+^ cells, including its CD4^+^ and CD8^+^ T cell subsets, and NK cell counts were significantly higher in subjects exposed to CS as compared to HC. Finally, we also observed a significant decrease of the total B cell count in each subgroup of CS-exposed subjects compared to HC. We did not find any difference between groups fort platelets, neutrophils, eosinophils and basophils counts.

### Reduction of Tregs Frequency With Duration of Silica Exposure

Circulating levels of Tregs have been reported decreased in several AID (25–32). We observed a significant decrease in the percentage of Tregs in subjects exposed to CS vs. HC (Figure 1B, left panel) (5.55% [4.61–6.72] vs. 6.81% [5.54–8.06], *p* = 0.02). When comparing percentages of Tregs among CS exposed workers, we observed that it significantly decreased between subgroups according to length of CS exposure (Figure 1B, right panel) (6.81% [5.54–8.06] vs. 6.03% [5.08–6.97], 5.88% [4.57–7.35], 5.38% [4.61–6.56] in not exposed, exposed <5 years, exposed 5–10 years, exposed >10 years, respectively; *p* = 0.046). We did not find any difference between groups when considering total Tregs counts (Figure 1C).

### Activated T Cells Increase in Workers With the Length of Silica Exposure

Because silica has been reported to behave as a super antigen (33), we next evaluated the expression of the activation marker (21, 22) HLA-DR CD3^+^, CD4^+^, and CD8^+^ T cells (Figure 2A). Complete data are reported in Supplemental Table 4.

Among CD3^+^ T cells, we observed a trend to an increase in the percentage of HLA-DR-expressing cells in CS exposed workers (*p* = 0.09, Figure 2B, left panel) and with length of exposure (Figure 2B, right panel). There was a significant increase of the total count of activated circulating CD3^+^ T cells (128.41 cells/μL [94.6–182.75] vs. 72.28 cells/μL [56.39–96.32] in HC, *p* = 0.0002, Figure 2C, left panel). This increase was mainly observed in the subgroups of subjects exposed 5–10 years (*p* = 0.001) or more than 10 years (*p* = 0.006, Figure 2C, right panel).

Similar observations were made when analyzing HLA-DR expression among CD3^+^CD4^+^ cells: it was increased in subject exposed to silica, whether considering its relative percentage, according to exposure status (*p* = 0.046, Figure 2B, left panel) and length of exposure (*p* = 0.057, Figure 2B, right panel) or especially considering its total count, according to exposure status and duration (*p* < 0.0001, Figure 2C). In the latter case, a significantly higher count of HLA-DR^+^CD4^+^ T cells was observed in each subgroup. In CD3^+^CD8^+^ cells, there was a significant increase in HLA-DR expression when comparing total counts according to exposure status (*p* = 0.004, Figure 2C, left panel) or exposure length (*p* = 0.012, Figure 2C, right panel).

### Decreased Tregs to Activated T Cells Ratio With Length of CS Exposure

**Figure 3:**
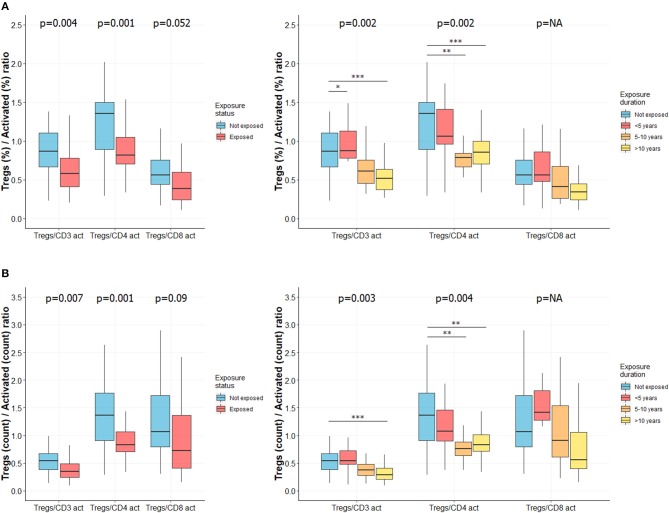
Ratio of Tregs to activated T cells is lower in CS exposed subjects and decreases with length of exposure. Ratio of Tregs to activated T cells was determined as described elsewhere (34). Ratio of percentages of Tregs to activated T cells in subjects exposed or not to silica (**A**, left) or in subgroups of subjects exposed to silica (**A**, right). Ratio of total count of Tregs to activated T cells in subjects exposed or not to silica (**B**, left) or in subgroups of subjects exposed to silica (**B**, right). Data are shown as boxplot. *P*-values represent Mann & Whitney test for two groups comparison and, Kruskal-Wallis test for four groups comparison (if previous test significant), followed by *post-hoc* Dunn test when applicable (results indicated by asterisks, *). *P*-values have been adjusted according to Hochberg method to account for repeated analysis. NA, not applicable.

We used the Tregs to activated T cells ratio to emphasize the relationship between these two subpopulations. The Tregs to activated CD3^+^ T cells ratio, as well as the Tregs to activated CD4^+^ T cells ratio were significantly decreased according to exposure status (Figure 3, left panels) and length of exposure (Figure 3, right panels). Differences were observed for both relative percentages (Figure 3A) and absolute cell counts (Figure 3B) (adjusted *p* < 0.01 for each analysis). Similar results were observed for Tregs to activated CD4^+^ T cell ratio, while only a trend was observed for Tregs to activated CD8^+^ T cell ratio (Figure 3). Complete data are reported in Supplemental Table 4.

### Higher Frequency of Autoantibodies Detection in CS Exposed Workers

**Figure 4:**
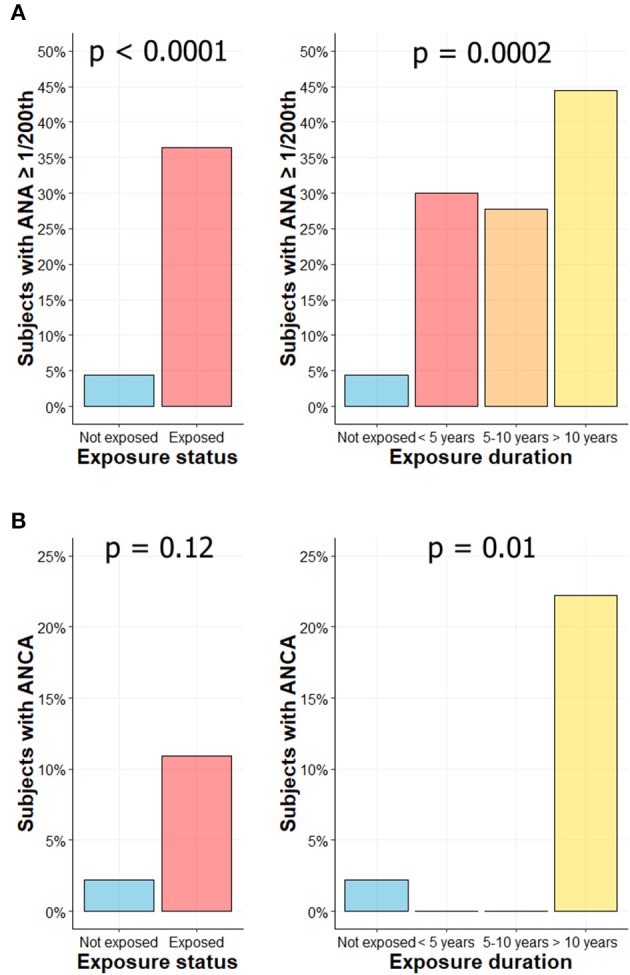
Higher frequency of autoantibodies levels in CS exposed workers. Percentage of subjects with ANA ≥ 1/200th according to exposure status (**A**, left) or exposure duration (**A**, right). Percentage of subjects with ANCA according to exposure status (**B**, left) or exposure duration (**B**, right).

ANA were found in 20/55 (36.4%) workers vs. in 2/45 (4.4%) HC (*p* < 0.0001; Figure 4A, left panel). Positive ANA was observed more frequently in subjects with longer CS exposure: 30% (3/10), 27.8% (5/18), and 44.4% (12/27) in subjects exposed <5 years, 5–10 years, and more than 10 years, respectively (*p* = 0.0002; Figure 4A, right panel). When sought (titer over 1/320th), no specificity was found for these ANAs and no anti-DNA antibodies were detected (data not shown). ANA titers are summarized in Supplemental Figure 2.

ANCA were found in 6/55 subjects (10.9%) exposed to CS vs. in 1/45 HC (2.2%) (*p* = 0.12; Figure 4B, left panel). The 6 ANCA-positive workers were exposed for more than 10 years to CS (6/27; 22.2%) (*p* = 0.01; Figure 4B, right panel). In exposed groups, the IIF pattern was cytoplasmic in 2 subjects (cANCA; titer 1/100th and 1/500th), perinuclear in 2 subjects (pANCA; titer 1/50th and 1/100th) and atypical in 2 subjects (aANCA; titers 1/500th). ANCA positivity was confirmed by ELISA in two subjects (one with a pANCA and one with an aANCA pattern), both exhibiting MPO reactivity. In control group, IIF pattern was cytoplasmic (titer 1/20th) without any identified target in ELISA. ACPA and RF were not detected in exposed workers.

## Discussion

In this prospective cohort study, we analyzed epidemiological, hematological and immunological data from 55 workers exposed to CS and free from silicosis. Compared to age- and sex- matched HC, we found a significant decrease in the frequency of Tregs and a significant increase in the absolute count of activated HLA-DR^+^ T-cell, associated with length of CS exposition. Moreover, we found a significant increase in ANA and ANCA frequency in CS exposed workers.

In contrast with our initial hypothesis, we did not find a decrease in total lymphocyte count in subject exposed to CS, but rather a slight increase during the first 10 years of CS exposure while remaining within the normal range. We suggest that this slight increase may be related to inflammation due to CS inhalation. Of note, previous studies that reported lymphopenia in silica-exposed workers, with (13) or without silicosis (35), enrolled much older subjects (70 ± 3.5 and 59 ± 10 years old, respectively) or much longer exposed workers (20 ± 9 years) (35). Anyway, we observed a significant decrease in the percentage of Tregs in our population. This could reflect the initial steps to tolerance breakdown, as a decrease in the frequency or number of Tregs has been associated with AID, such as SLE (28, 29), systemic sclerosis (25–27) or rheumatoid arthritis (30–32). In AAV patients, unlike other AID, an increased proportion of Tregs has rather been reported (36) but with impaired properties (37). An imbalance between effector and regulatory T cells has been demonstrated critical in the pathogenesis of these diseases.

This is, to our knowledge, the first study showing a decreased percentage of Tregs in workers exposed to CS free from silicosis and from any other AID. In silicosis patients, two studies showed that Tregs were decreased. However, a limitation in these studies is that Tregs were defined as CD4^+^CD25^+^ cells and FoxP3 expression was not assessed (38, 39). Moreover, when PBMC from HC were cultured with silica, CD4^+^FoxP3^+^ cells was markedly decreased (40).

In patients with silicosis, different mechanisms have been proposed to explain changes in Tregs/effector T cells balance. First, an increase in Tregs apoptosis has been related to and increased expression of the cell death receptor CD95/Fas molecule (41), and to anti-Fas antibodies (42). Second, different signals lead to the activation and proliferation of T helper cell: they express more PD-1 (activation marker) (40) and Decoy Receptor 3 (DcR3, which prevents Fas-induced apoptosis) (43). They also produce a soluble form of the CD95, which is anti-apoptotic and release more soluble IL-2 receptor (42, 44). Thus, this imbalance may sustain the effector functions of lymphocytes. Another explanation for Tregs decrease in peripheral blood, is that they may be recruited into secondary lymphoid organs or subclinical damaged organs such as lungs. Indeed, Huaux et al. reported that Tregs were markedly accumulated in the lung and the thymus during the development of silica-induced lung fibrosis in mice (45).

Secondly, through an increased expression of HLA-DR, we found a more pronounced activation profile in T cells, including total CD3^+^, CD4^+^, and CD8^+^ T cells. The HLA-DR antigen is a cell surface glycoprotein encoded by genes of the major histocompatibility complex. It is constitutively expressed on the surface of antigen presenting cells (monocytes, macrophages, B cells) and is normally absent on resting T cells. However, it can be found at the cell surface of a significant percentage of T cells upon activation. More precisely, it appears at late stages of T cells activation: its expression starts after 24 h of activation and remains high for up to several weeks (46). It is thus considered as a very late activation marker, allowing to evaluate the global activation status of immune cells. Its expression on T cells was proven to correlate with lupus activity either when analyzed on CD3^+^ T cells (47), CD4^+^ T cells (22, 48–52), or CD8^+^ T cells (22, 49, 51, 52). It was shown that HLA-DR expression on CD3^+^ T cells was higher in lupus patients with anti-DNA antibodies as compared to HC (47) and was higher on CD8^+^ T cells in patients experiencing a lupus flare (22). Similar observations were made in patients with rheumatoid arthritis (21, 53), antiphospholipid syndrome (54) and with rejection in solid organ transplantation (55).

The increased percentage of activated T cells that we found in subjects exposed to silica could reflect a chronic T cells stimulation by silica particles released from damaged macrophages. Indeed, *in vitro*, silica can have a “superantigen” action on human T cells (33). In other words, it means that silica can lead to the activation of a T cell by an antigen presenting cell (APC) regardless of its specificity for the presented antigen. Thus, silica particles have an adjuvant effect that can non-specifically enhance the immune response. However, the threshold above which this long-established adjuvant effect appears is not yet fully understood (56) and remains to be studied.

This activation status observed in subjects exposed to CS is consistent with some previous reports. Indeed, increased levels of circulating soluble IL-2 receptor, released by activated T cells after cleavage of its membrane form, were found in subjects exposed to silica, with (44) or without silicosis (57). We also join back our previous data showing an increased HLA-DR expression on total CD3^+^ T cells in silicotic patients (13). Finally, our results are also congruent with *in vitro* findings: first, the expression of the early expression marker CD69 was increased on lymphocytes when PBMC were cultured with silica (58); second, it was reported that silica could have a direct effect on T and B cells activation (activation of the TCR-complex and of the BCR-complex indicated by an increased phosphorylation level of the zeta chain and Igα, respectively) and proliferation (indicated by an increased expression of the transcription factor c-Myc), demonstrating that APC are not essential in these pathways and that silica can circumvents many self-tolerance check-points (59).

The ratio between Tregs and activated T cells, which reflect the imbalance between both subsets (34), was significantly decreased (especially Tregs to activated CD3^+^ and CD4^+^ T cells) in exposed workers and decreased along with duration of CS exposure. Thus, it may be used to identify patients at risk of tolerance breakdown; however, this needs to be specifically evaluated.

Whether considering Tregs, activated T cells or their ratio, results found in the group with the shortest time of exposure were often close to those found in the control group, and gradually increased (or decreased) with exposure duration. This suggests a coherent cumulative effect of silica exposure duration. This has already been underlined in the cohort of World Trade Center–exposed firefighters and Emergency Medical Service workers (60). In this cohort, it was demonstrated that every additional month worked in the dusty site increased the occurrence of AID.

At last, we highlighted an increased frequency of auto-antibodies in subjects exposed to CS. ANA and ANCA were particularly more frequent in the subgroup with the longer exposure to CS. ANA and ANCA have been mainly found in ill silica-exposed workers, with silicosis (13, 61, 62) or AID alone (10, 63). However, our results are consistent with others studies including asymptomatic exposed subjects (56, 62, 64, 65). This result is also in accordance with animal data showing an increased production of ANA in lupus-prone NZM mice (66) and the known adjuvant effect of CS on antibody production (67). However, it is unclear whether autoantibody production in silica-exposed workers is predictive for the development of further AID. In opposition to some studies, our results and another study (68) did not detect RF nor ACPA in CS exposed workers. We suggest that these discrepancies may be related to physical particularities of CS microparticles in our cohort.

The major limitation of our study is that we could not identify precisely the duration of exposure, e.g., subjects exposed during 20 years to CS were pooled with those exposed only 11 years in the same subgroups, and, in opposition, subjects exposed only a few months were pooled with those exposed 4 years. This pooling strategy may have underestimated or overestimated some of the differences observed. Another limitation is that we did not follow subjects exposed to CS over time and could not correlate our findings with further occurrence of AID. Lastly, beyond silica exposure on regulatory T cells and T cells activation, many other immune tolerance mechanisms were not investigated. For example, we did not examine HLA alleles, nor did we study the link between potential MHC Class II restrictions and autoantibodies development.

In conclusion, we show that silica exposure is associated with a tolerance breakdown. It is associated with a decrease of Tregs frequency and an increase of T cell activation, and, consequently, an imbalance of Tregs/activated T cells ratio. We hypothesize that this leads to B cell activation leading ultimately to the production of auto-antibodies. These immune dysregulations are already present in non-silicotic CS-exposed subjects. Whether silicosis represent a later stage of these dysregulations or if specific endogenous or exogenous factors lead to silicosis development remain to be studied.

**Figure 5:**
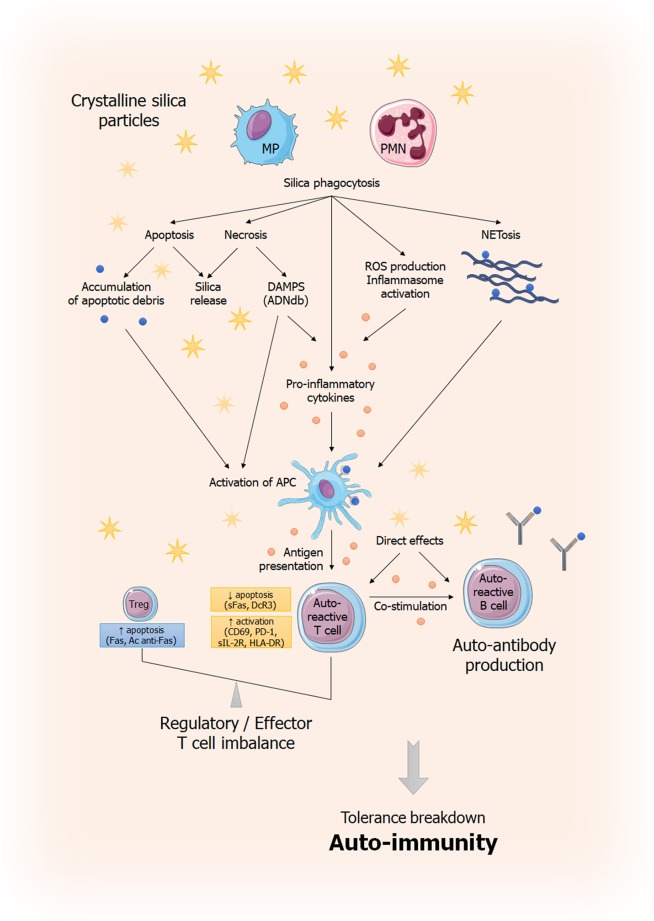
General overview of immune dysregulation induced by silica exposure. This figure summarizes current understanding of silica-induced tolerance breakdown. Phagocytosis of silica particles leads to inflammasome activation (69, 70), production of pro-inflammatory cytokines (IL-1β, TNF-α) (71, 72) and ROS production (71, 73, 74). Phagocytic cells capable of antigen presentation (APC) will induce an effector T response amplified by the dysregulation of the effector/regulator T balance and resulting to autoantibodies secretion by B cells. This imbalance is the result, on the one hand, of anti-apoptotic signals (sFas, DcR3) (42, 43) and, on the other hand, pro-apoptotic signals (increased Fas expression, anti-Fas antibodies) (42). Silica particles are also believed to play a direct role in lymphocyte activation and proliferation. In parallel, cells that have phagocyted silica, unable to destroy it, will not only release it (thus perpetuating the phenomenon) but also will die, releasing DAMPs (in case of necrosis) or responsible of apoptotic debris accumulation, also leading to inappropriate activation of the immune system (75). Ag, antigen; APC: antigen presenting cell; DAMP: Danger Associated Molecular Pattern; MP; macrophages; PMN, polymorphonuclear; ROS, Reactive Oxygen Species; sFas: soluble Fas; DcR3: Decoy Receptor 3.

These mechanisms and others previously described, that lead to auto-immunity and tissue damage, are summarized in Figure 5.

## Data Availability Statement

The datasets generated for this study are available on request to the corresponding author.

## Ethics Statement

The studies involving human participants were reviewed and approved by Ethical committee of Angers University Hospital (CCPPRB 2006/23 and 2006/23 bis). The patients/participants provided their written informed consent to participate in this study.

## Author Contributions

PR, YR, PJ, GR, J-FS, and J-FA contributed to the conception and design of the study. JT, GR, and J-FA supervised the project. BB, GM, and GR organized the database. BB and JR performed the statistical analysis. BB wrote the first draft of the manuscript. J-FA provided critical revision of the manuscript. All authors read and approved the submitted version.

### Conflict of Interest

The authors declare that the research was conducted in the absence of any commercial or financial relationships that could be construed as a potential conflict of interest.

## Supplementary Material

Supplemental Figure 1 Flowchart of the study.

Supplemental Figure 2 Antinuclear antibody (ANA) titers. ANA titers according to silica exposure status **(A)** or exposure duration **(B)**. Each dot represents one subject. ND, not detected.

Supplemental Table 1 Antibodies used for flow cytometry analysis.

Supplemental Table 2 Occupation of subjects exposed to silica (*n* = 55).

Supplemental Table 3 Hematological data. Data are show as Mean ± SD or Median [interquartile range]. Statistical tests have been performed sequentially: (1) Mann & Whitney test (otherwise specified), (2) Kruskal-Wallis test (otherwise specified), if previous test significant, (3) Dunn *post-hoc* test, if previous test significant (0 = control group, 1,2,3 = groups exposed <5, 5–10, >10 years, respectively). *P*-values have been adjusted according to Hochberg method to account for repeated analysis.

Supplemental Table 4 Regulatory T cells (Tregs), activated T cells and ratio of Tregs to activated cells, complete data. Data are shown as Median [Interquartile range]. Count is in cells/μL. Statistical tests have been performed sequentially: (1) Mann & Whitney test, (2) Kruskal-Wallis test, if previous test significant, (3) Dunn *post-hoc* test, if previous test significant (0 = control group, 1,2,3 = groups exposed <5, 5–10, >10 years, respectively). *P*-values have been adjusted according to Hochberg method to account for repeated analysis.

## Abbreviations

AAV: ANCA Associated Vasculitis
ACPA: Anti-Citrullinated Protein Antibody
AID: Autoimmune Disease
ANA: Antinuclear Antibodies
ANCA: Anti-Neutrophil Cytoplasmic Antibodies
ANOVA: Analysis of Variance
APC: Antigen Presenting Cell
CD: Cluster of Differentiation
COPD: Chronic obstructive pulmonary disease
CS: Crystalline Silica
HLA: Human Leucocyte Antigen
IIF: Indirect ImmunoFluorescence
IQR: Interquartile Range
MEV: Mean Exposure Value
PBMC: Peripheral Blood Mononuclear Cell
RA: Rheumatoid Arthritis
ROS: Reactive Oxygen Species
SLE: Systemic Lupus Erythematosus
SSc: Systemic Sclerosis
Tregs: regulatory T cells.

## References

[1] Rudnick RL Holland HD (eds). The Crust. 1st ed. Amsterdam: Elsevier (2005).

[2] Leung CC Yu ITS Chen W. Silicosis. Lancet Lond Engl. (2012) 379:2008–18. 10.1016/S0140-6736(12)60235-9 22534002

[3] Khuder SA Peshimam AZ Agraharam S. Environmental risk factors for rheumatoid arthritis. Rev Environ Health. (2002) 17:307–15. 10.1515/REVEH.2002.17.4.307 12611472

[4] Stolt P Källberg H Lundberg I Sjögren B Klareskog L Alfredsson L. Silica exposure is associated with increased risk of developing rheumatoid arthritis: results from the Swedish EIRA study. Ann Rheum Dis. (2005) 64:582–6. 10.1136/ard.2004.022053 15319232 PMC1755463

[5] McCormic ZD Khuder SS Aryal BK Ames AL Khuder SA. Occupational silica exposure as a risk factor for scleroderma: a meta-analysis. Int Arch Occup Environ Health. (2010) 83:763–9. 10.1007/s00420-009-0505-7 20047060

[6] Parks CG Cooper GS Nylander-French LA Sanderson WT Dement JM Cohen PL. Occupational exposure to crystalline silica and risk of systemic lupus erythematosus: a population-based, case-control study in the Southeastern United States. Arthritis Rheum. (2002) 46:1840–50. 10.1002/art.10368 12124868

[7] Cooper GS Wither J Bernatsky S Claudio JO Clarke A Rioux JD. Occupational and environmental exposures and risk of systemic lupus erythematosus: silica, sunlight, solvents. Rheumatology. (2010) 49:2172–80. 10.1093/rheumatology/keq214 20675707 PMC2954367

[8] Makol A Reilly MJ Rosenman KD. Prevalence of connective tissue disease in silicosis (1985-2006)-a report from the state of michigan surveillance system for silicosis. Am J Ind Med. (2011) 54:255–62. 10.1002/ajim.20917 20957678

[9] Conrad K Mehlhorn J Lüthke K Dörner T Frank KH. Systemic lupus erythematosus after heavy exposure to quartz dust in uranium mines: clinical and serological characteristics. Lupus. (1996) 5:62–9. 10.1177/096120339600500112 8646229

[10] Hogan SL Satterly KK Dooley MA Nachman PH Jennette JC Falk RJ. Silica exposure in anti-neutrophil cytoplasmic autoantibody-associated glomerulonephritis and lupus nephritis. J Am Soc Nephrol. (2001) 12:134–42. 11134259 10.1681/ASN.V121134

[11] Lane SE Watts RA Bentham G Innes NJ Scott DGI. Are environmental factors important in primary systemic vasculitis?: A case-control study. Arthritis Rheum. (2003) 48:814–23. 10.1002/art.10830 12632437

[12] Hogan SL Cooper GS Savitz DA Nylander-French LA Parks CG Chin H. Association of silica exposure with anti-neutrophil cytoplasmic autoantibody small-vessel vasculitis: a population-based, case-control study. Clin J Am Soc Nephrol. (2007) 2:290–9. 10.2215/CJN.03501006 17699427 PMC4049534

[13] Subra JF Renier G Reboul P Tollis F Boivinet R Schwartz P Lymphopenia in occupational pulmonary silicosis with or without autoimmune disease. Clin Exp Immunol. (2001) 126:540–4. 10.1046/j.1365-2249.2001.01696.x 11737074 PMC1906221

[14] Watanabe S Shirakami A Takeichi T Ohara T Saito S. Alterations in lymphocyte subsets and serum immunoglobulin levels in patients with silicosis. J Clin Lab Immunol. (1987) 23:45–51. 3612759

[15] Vincent R Jeandel B. COLCHIC - occupational exposure to chemical agents database: current content and development perspectives. Appl Occup Environ Hyg. (2001) 16:115–21. 10.1080/104732201460190 11217697

[16] Mater G Paris C Lavoué J. Descriptive analysis and comparison of two French occupational exposure databases: COLCHIC and SCOLA: comparison of two French occupational exposure databases. Am J Ind Med. (2016) 59:379–91. 10.1002/ajim.22569 26901238

[17] Delabre L Pilorget C Garras L Févotte J et le Groupe Matgéné. Éléments Techniques sur l'Exposition Professionnelle aux Poussières Alvéolaires de Silice Cristalline Libre - Présentation d'une Matrice Emplois-Expositions aux Poussières Alvéolaires de Silice Cristalline Libre. Saint-Maurice: Institut de Veille Sanitaire, Février (2010). p. 15 Available online at: https://www.santepubliquefrance.fr/ (accessed June 15, 2019).

[18] Agmon-Levin N Damoiseaux J Kallenberg C Sack U Witte T Herold M. International recommendations for the assessment of autoantibodies to cellular antigens referred to as anti-nuclear antibodies. Ann Rheum Dis. (2014) 73:17–23. 10.1136/annrheumdis-2013-203863 24126457

[19] Dragon-Durey M-A Fabien N Chyderiotis G Musset L Pham B-N Olsson N. Testing anti-neutrophil cytoplasmic antibodies (ANCA): analysis of the European EASI survey on the daily practice of the French laboratories. Ann Biol Clin. (2017) 75:531–41. 10.1684/abc.2017.1273 28958962

[20] Santegoets SJAM Dijkgraaf EM Battaglia A Beckhove P Britten CM Gallimore A. Monitoring regulatory T cells in clinical samples: consensus on an essential marker set and gating strategy for regulatory T cell analysis by flow cytometry. Cancer Immunol Immunother. (2015) 64:1271–86. 10.1007/s00262-015-1729-x 26122357 PMC4554737

[21] Pincus SH Clegg DO Ward JR. Characterization of T cells bearing HLA-DR antigens in rheumatoid arthritis. Arthritis Rheum. (1985) 28:8–15. 10.1002/art.1780280103 3917673

[22] Viallard JF Bloch-Michel C Neau-Cransac M Taupin JL Garrigue S Miossec V. HLA-DR expression on lymphocyte subsets as a marker of disease activity in patients with systemic lupus erythematosus. Clin Exp Immunol. (2001) 125:485–91. 10.1046/j.1365-2249.2001.01623.x 11531958 PMC1906149

[23] Lafaye de Micheaux P Liquet B Marque S Riou J. Power and sample size determination in clinical trials with multiple primary continuous correlated endpoints. J Biopharm Stat. (2014) 24:378–97. 10.1080/10543406.2013.860156 24605975

[24] Bretz F Hothorn T Westfall P Multiple Comparisons Using R. New York, NY: Chapman and Hall/CRC (2016). 10.1201/9781420010909

[25] Frantz C Auffray C Avouac J Allanore Y. Regulatory T cells in systemic sclerosis. Front Immunol. (2018) 9:2356. 10.3389/fimmu.2018.02356 30374354 PMC6196252

[26] Slobodin G Rimar D. Regulatory T cells in systemic sclerosis: a comprehensive review. Clin Rev Allergy Immunol. (2017) 52:194–201. 10.1007/s12016-016-8563-6 27318947

[27] Ujiie H. Regulatory T cells in autoimmune skin diseases. Exp Dermatol. (2018) 28:642–6. 10.1111/exd.13535 29575350

[28] Zhu Y Huang Y Ming B Wu X Chen Y Dong L. Regulatory T-cell levels in systemic lupus erythematosus patients: a meta-analysis. Lupus. (2019) 28:445–54. 10.1177/0961203319828530 30744525

[29] Li W Deng C Yang H Wang G. The regulatory T cell in active systemic lupus erythematosus patients: a systemic review and meta-analysis. Front Immunol. (2019) 10:159. 10.3389/fimmu.2019.00159 30833946 PMC6387904

[30] Zhang X Zhang X Zhuang L Xu C Li T Zhang G. Decreased regulatory T-cell frequency and interleukin-35 levels in patients with rheumatoid arthritis. Exp Ther Med. (2018) 16:5366–72. 10.3892/etm.2018.6885 30542496 PMC6257791

[31] Yang M Liu Y Mo B Xue Y Ye C Jiang Y Helios but not CD226, TIGIT and Foxp3 is a potential marker for CD4+ Treg cells in patients with rheumatoid arthritis. Cell Physiol Biochem. (2019) 52:1178–92. 10.33594/000000080 30990587 PMC6943339

[32] Nakano S Morimoto S Suzuki S Tsushima H Yamanaka K Sekigawa I. Immunoregulatory role of IL-35 in T cells of patients with rheumatoid arthritis. Rheumatol Oxf Engl. (2015) 54:1498–506. 10.1093/rheumatology/keu528 25731770

[33] Ueki A Yamaguchi M Ueki H Watanabe Y Ohsawa G Kinugawa K. Polyclonal human T-cell activation by silicate *in vitro*. Immunology. (1994) 82:332–5. 7927506 PMC1414818

[34] Carter CRD Aravind G Smalle NL Cole JY Savic S Wood PMD. CVID patients with autoimmunity have elevated T cell expression of granzyme B and HLA-DR and reduced levels of Treg cells. J Clin Pathol. (2013) 66:146–50. 10.1136/jclinpath-2012-201046 23172556

[35] Rocha MC Santos LMB Bagatin E Tervaert JWC Damoiseaux JGMC Lido AV. Genetic polymorphisms and surface expression of CTLA-4 and PD-1 on T cells of silica-exposed workers. Int J Hyg Environ Health. (2012) 215:562–9. 10.1016/j.ijheh.2011.10.010 22153879

[36] Abdulahad WH Stegeman CA van der Geld YM Doornbos-van der Meer B Limburg PC Kallenberg CGM. Functional defect of circulating regulatory CD4+ T cells in patients with Wegener's granulomatosis in remission. Arthritis Rheum. (2007) 56:2080–91. 10.1002/art.22692 17530650

[37] Morgan MD Day CJ Piper KP Khan N Harper L Moss PA. Patients with Wegener's granulomatosis demonstrate a relative deficiency and functional impairment of T-regulatory cells. Immunology. (2010) 130:64–73. 10.1111/j.1365-2567.2009.03213.x 20113371 PMC2855794

[38] Wu P Miura Y Hyodoh F Nishimura Y Hatayama T Hatada S. Reduced function of CD4+25+ regulatory T cell fraction in silicosis patients. Int J Immunopathol Pharmacol. (2006) 19:357–68. 10.1177/039463200601900212 16831302

[39] Carlsten C de Roos AJ Kaufman JD Checkoway H Wener M Seixas N. Cell markers, cytokines, and immune parameters in cement mason apprentices. Arthritis Rheum. (2007) 57:147–53. 10.1002/art.22483 17266079

[40] Hayashi H Miura Y Maeda M Murakami S Kumagai N Nishimura Y. Reductive alteration of the regulatory function of the CD4(+)CD25(+) T cell fraction in silicosis patients. Int J Immunopathol Pharmacol. (2010) 23:1099–109. 10.1177/039463201002300414 21244759

[41] Strasser A Jost PJ Nagata S. The many roles of FAS receptor signaling in the immune system. Immunity. (2009) 30:180–92. 10.1016/j.immuni.2009.01.001 19239902 PMC2956119

[42] Otsuki T Hayashi H Nishimura Y Hyodo F Maeda M Kumagai N. Dysregulation of autoimmunity caused by silica exposure and alteration of Fas-mediated apoptosis in T lymphocytes derived from silicosis patients. Int J Immunopathol Pharmacol. (2011) 24:11S−16S. 21329560

[43] Otsuki T Tomokuni A Sakaguchi H Aikoh T Matsuki T Isozaki Y. Over-expression of the decoy receptor 3 (DcR3) gene in peripheral blood mononuclear cells (PBMC) derived from silicosis patients. Clin Exp Immunol. (2000) 119:323–7. 10.1046/j.1365-2249.2000.01132.x 10632670 PMC1905509

[44] Hayashi H Maeda M Murakami S Kumagai N Chen Y Hatayama T. Soluble interleukin-2 receptor as an indicator of immunological disturbance found in silicosis patients. Int J Immunopathol Pharmacol. (2009) 22:53–62. 10.1177/039463200902200107 19309552

[45] Huaux F. [A new pathologic pathway for pulmonary fibrosis induced by silica: involvement of immunosuppressive responses]. Bull Mem Acad R Med Belg. (2009) 164:240–6. 20666153

[46] Pichler WJ Wyss-Coray T. T cells as antigen-presenting cells. Immunol Today. (1994) 15:312–5. 10.1016/0167-5699(94)90078-7 7522009

[47] Zhou H Li B Li J Wu T Jin X Yuan R. Dysregulated T cell activation and aberrant cytokine expression profile in systemic lupus erythematosus. Mediators Inflamm. (2019) 2019:8450947. 10.1155/2019/8450947 31007604 PMC6441516

[48] Daca A Czuszynska Z Smolenska Z Zdrojewski Z Witkowski JM Bryl E. Two systemic lupus erythematosus (SLE) global disease activity indexes–the SLE Disease Activity Index and the Systemic Lupus Activity Measure–demonstrate different correlations with activation of peripheral blood CD4+ T cells. Hum Immunol. (2011) 72:1160–7. 10.1016/j.humimm.2011.08.005 21906646

[49] Wenzel J Henze S Brähler S Bieber T Tüting T. The expression of human leukocyte antigen-DR and CD25 on circulating T cells in cutaneous lupus erythematosus and correlation with disease activity. Exp Dermatol. (2005) 14:454–9. 10.1111/j.0906-6705.2005.00301.x 15885081

[50] Watanabe T Suzuki J Mitsuo A Nakano S Tamayama Y Katagiri A Striking alteration of some populations of T/B cells in systemic lupus erythematosus: relationship to expression of CD62L or some chemokine receptors. Lupus. (2008) 17:26–33. 10.1177/0961203307085246 18089680

[51] Shirota Y Yarboro C Fischer R Pham T-H Lipsky P Illei GG. Impact of anti-interleukin-6 receptor blockade on circulating T and B cell subsets in patients with systemic lupus erythematosus. Ann Rheum Dis. (2013) 72:118–28. 10.1136/annrheumdis-2012-201310 22858586

[52] Edelbauer M Kshirsagar S Riedl M Billing H Tönshoff B Haffner D. Activity of childhood lupus nephritis is linked to altered T cell and cytokine homeostasis. J Clin Immunol. (2012) 32:477–87. 10.1007/s10875-011-9637-0 22228566

[53] Kluin-Nelemans HC van der Linden JA Gmelig Meyling FH Schuurman HJ. HLA-DR positive T lymphocytes in blood and synovial fluid in rheumatoid arthritis. J Rheumatol. (1984) 11:272–6. 6610750

[54] Carbone J Gallego A Lanio N Navarro J Orera M Aguaron A. Quantitative abnormalities of peripheral blood distinct T, B, and natural killer cell subsets and clinical findings in obstetric antiphospholipid syndrome. J Rheumatol. (2009) 36:1217–25. 10.3899/jrheum.081079 19332638

[55] Jung HY Kim YJ Choi JY Cho JH Park SH Kim YL. Increased circulating T lymphocytes expressing HLA-DR in kidney transplant recipients with microcirculation inflammation. J Korean Med Sci. (2017) 32:908–18. 10.3346/jkms.2017.32.6.908 28480647 PMC5426246

[56] Parks CG Conrad K Cooper GS. Occupational exposure to crystalline silica and autoimmune disease. Environ Health Perspect. (1999) 107(Suppl. 5):793–802. 10.1289/ehp.99107s5793 10970168 PMC1566238

[57] Rocha-Parise M Santos LMB Damoiseaux JGMC Bagatin E Lido AV Torello CO. Lymphocyte activation in silica-exposed workers. Int J Hyg Environ Health. (2014) 217:586–91. 10.1016/j.ijheh.2013.11.002 24332681

[58] Wu P Hyodoh F Hatayama T Sakaguchi H Hatada S Miura Y Induction of CD69 antigen expression in peripheral blood mononuclear cells on exposure to silica, but not by asbestos/chrysotile-A. Immunol Lett. (2005) 98:145–52. 10.1016/j.imlet.2004.11.005 15790520

[59] Eleftheriadis T Pissas G Zarogiannis S Liakopoulos V Stefanidis I. Crystalline silica activates the T-cell and the B-cell antigen receptor complexes and induces T-cell and B-cell proliferation. Autoimmunity. (2019) 52:136–43. 10.1080/08916934.2019.1614171 31119949

[60] Webber MP Moir W Crowson CS Cohen HW Zeig-Owens R Hall CB. Post–September 11, 2001, incidence of systemic autoimmune diseases in world trade center–exposed firefighters and emergency medical service workers. Mayo Clin Proc. (2016) 91:23–32. 10.1016/j.mayocp.2015.09.019 26682920 PMC4968872

[61] Lee S Hayashi H Mastuzaki H Kumagai-Takei N Otsuki T Silicosis and autoimmunity: *Curr Opin Allergy Clin Immunol*. (2017) 17:78–84. 10.1097/ACI.0000000000000350 28177948

[62] Bartunková J Pelclová D Fenclová Z Šedivá A Lebedová J Tesar V. Exposure to silica and risk of ANCA-associated vasculitis. Am J Ind Med. (2006) 49:569–76. 10.1002/ajim.20327 16691610

[63] Gregorini G Ferioli A Donato F Tira P Morassi L Tardanico R. Association between silica exposure and necrotizing crescentic glomerulonephritis with p-ANCA and anti-MPO antibodies: a hospital-based case-control study. Adv Exp Med Biol. (1993) 336:435–40. 10.1007/978-1-4757-9182-2_77 8296651

[64] Conrad K Mehlhorn J. Diagnostic and prognostic relevance of autoantibodies in uranium miners. Int Arch Allergy Immunol. (2000) 123:77–91. 10.1159/000024426 11014974

[65] Conrad K Stahnke G Liedvogel B Mehlhorn J Barth J Blasum C. Anti-CENP-B response in sera of uranium miners exposed to quartz dust and patients with possible development of systemic sclerosis (scleroderma). J Rheumatol. (1995) 22:1286–94. 7562760

[66] Brown JM Archer AJ Pfau JC Holian A. Silica accelerated systemic autoimmune disease in lupus-prone New Zealand mixed mice. Clin Exp Immunol. (2003) 131:415–21. 10.1046/j.1365-2249.2003.02094.x 12605693 PMC1808650

[67] Pernis B Paronetto F. Adjuvant effect of silica (Tridymite) on antibody production. Exp Biol Med. (1962) 110:390–2. 10.3181/00379727-110-27527 14485446

[68] Aminian O Sharifian SA Mehrdad R Haghighi KS Mazaheri M. Antinuclear antibody and rheumatoid factor in silica-exposed workers. Arh Hig Rada Toksikol. (2009) 60:185–90. 10.2478/10004-1254-60-2009-1892 19581212

[69] Dostert C Pétrilli V Van Bruggen R Steele C Mossman BT Tschopp J. Innate immune activation through Nalp3 inflammasome sensing of asbestos and silica. Science. (2008) 320:674–7. 10.1126/science.1156995 18403674 PMC2396588

[70] Hornung V Bauernfeind F Halle A Samstad EO Kono H Rock KL. Silica crystals and aluminum salts activate the NALP3 inflammasome through phagosomal destabilization. Nat Immunol. (2008) 9:847–56. 10.1038/ni.1631 18604214 PMC2834784

[71] Kawasaki H. A mechanistic review of silica-induced inhalation toxicity. Inhal Toxicol. (2015) 27:363–77. 10.3109/08958378.2015.1066905 26194035

[72] Rabolli V Lison D Huaux F. The complex cascade of cellular events governing inflammasome activation and IL-1β processing in response to inhaled particles. Part Fibre Toxicol. (2015) 13:40. 10.1186/s12989-016-0150-8 27519871 PMC4983011

[73] Fubini B Fenoglio I Elias Z Poirot O. Variability of biological responses to silicas: effect of origin, crystallinity, and state of surface on generation of reactive oxygen species and morphological transformation of mammalian cells. J Environ Pathol Toxicol Oncol. (2001) 20(Suppl. 1):95–108. 10.1615/JEnvironPatholToxicolOncol.v20.iSuppl.1.90 11570678

[74] Fubini B Hubbard A. Reactive oxygen species (ROS) and reactive nitrogen species (RNS) generation by silica in inflammation and fibrosis. Free Radic Biol Med. (2003) 34:1507–16. 10.1016/S0891-5849(03)00149-7 12788471

[75] Brown JM Pfau JC Pershouse MA Holian A. Silica, apoptosis, and autoimmunity. J Immunotoxicol. (2005) 1:177–87. 10.1080/15476910490911922 18958651

