# Supplementary figures and images for: T Cell Dysregulation in Non-silicotic Silica Exposed Workers: A Step Toward Immune Tolerance Breakdown
Source: Front Immunol. 2019 Nov 22;10:2743. doi: 10.3389/fimmu.2019.02743 (PMC6883424; doi:10.3389/fimmu.2019.02743)

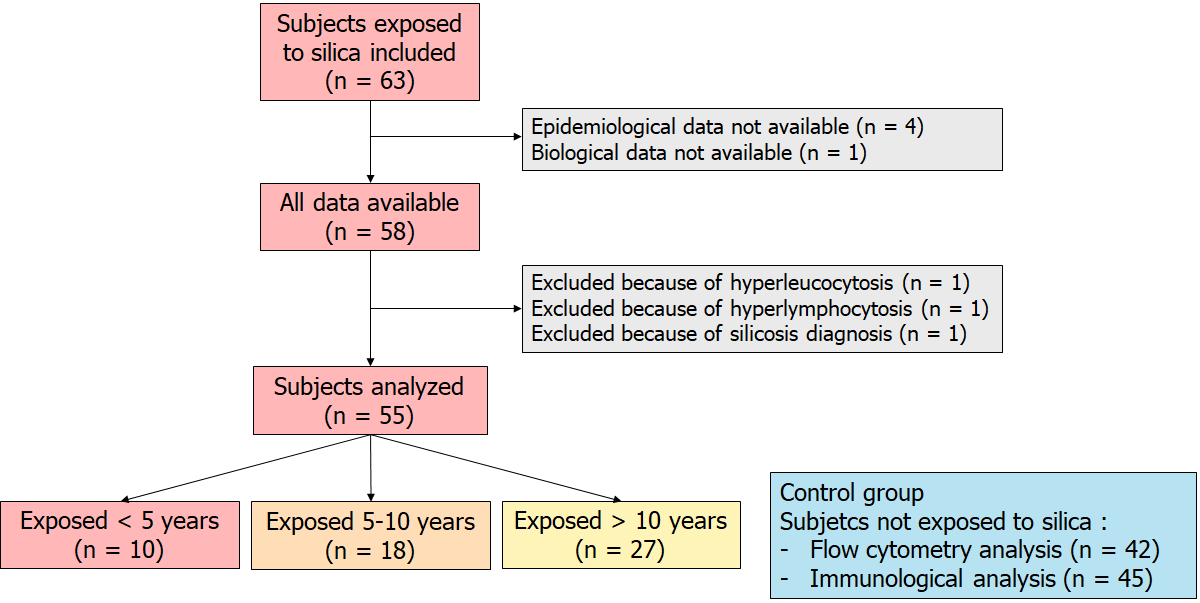

Supplement: Supplemental Figure 1 — Flowchart of the study. [file Image_1.tif]

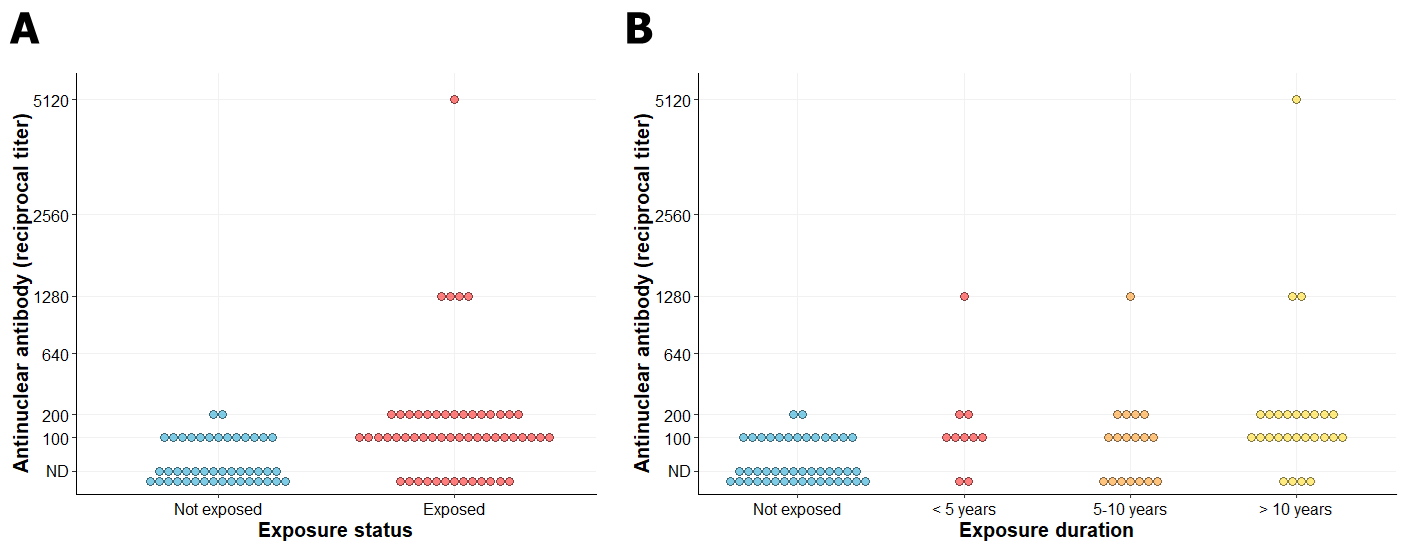

Supplement: Supplemental Figure 2 — Antinuclear antibody (ANA) titers. ANA titers according to silica exposure status (A) or exposure duration (B). Each dot represents one subject. ND, not detected. [file Image_2.tif]
