# Supplementary material for: T Cell Dysregulation in Non-silicotic Silica Exposed Workers: A Step Toward Immune Tolerance Breakdown
Source: Front Immunol. 2019 Nov 22;10:2743. doi: 10.3389/fimmu.2019.02743 (PMC6883424; doi:10.3389/fimmu.2019.02743)
Supplement: Supplemental Table 1 — Antibodies used for flow cytometry analysis. [file Data_Sheet_1.pdf]

Supplemental Table 1. Antibodies used for flow cytometry analysis.

Target	Fluorochrome	Clone	Isotype	Supplier	Cat #
**Regulatory T cells**					
CD3	PerCP-Cy5	SK7	mouse IgG1,κ	BD Bioscience	332771
CD4	Pacific Blue	RPA-T4	mouse IgG1,κ	BD Bioscience	558116
CD8	Alexa 700	RPA-T8	mouse IgG1,κ	BD Bioscience	557945
CD25	FITC	2A3	mouse IgG1,κ	BD Bioscience	345796
CD127	PE	hIL-7R-M21	mouse IgG1,κ	BD Bioscience	557938
FoxP3	APC	PCH101	rat IgG2a,κ	eBiosciences	17-4776-71
**Lymphocyte activation**					
CD3	PerCP-Cy5	SK7	mouse IgG1,κ	BD Bioscience	332771
CD4/CD8	FITC/PE	n. a.	mouse IgG1	Beckman Coulter	6604614
HLA-DR	APC	G46-6	mouse IgG2a,κ	BD Bioscience	559866
**Lymphocyte subtype (T cells, B cells, NK cells)**					
CD19	APC	SJ25C1	mouse IgG1,κ	BD Bioscience	345791
CD3/CD16+56	FITC/PE	n. a.	mouse IgG1	BD Bioscience	342403

Supplemental Table 2. Occupation of subjects exposed to silica (n = 55).

Occupation	n
Manufacturing team leader	10
Truck driver	7
Machine operator	7
Laboratory technician	6
Production agent	6
Maintenance agent	4
Warehouseman	4
Order processor	4
Manager	4
Colorist	2
Cleaning agent	1

Supplemental Table 3. Hematological data.

	Normal range	Not exposed to CS (n = 42)	All exposed subjects (n = 55)	Adjusted p-values (not exposed vs exposed)	Exposed < 5 years (n = 10)	Exposed 5-10 years (n = 18)	Exposed > 10 years (n = 27)	Adjusted p-values (4 groups comparisons)	Significant intergroup comparison
Hemoglobin (g/dl)	13,5-17,5	14,91 ± 0,93	15,74 ± 0,90	**p < 0.0001** (T test)	15,52 ± 0,87	15,78 ± 0,86	15,80 ± 0,95	**0.004** (ANOVA)	0-2, 0-3
Platelets (G/L)	150-400	244 ± 60	246 ± 54	0.92 (T test)	245 ± 51	250 ± 64	244 ± 51	-	-
Leucocytes (G/L)	4-10	5.40 [4.61-5.86]	6.24 [5.03-7.45]	**0.02**	5.91 [5.05-8.36]	6.82 [5.69-7.48]	5.70 [4.74-6.78]	**0.018**	0-2
Lymphocytes (G/L)	1-4,5	1.71 [1.39-2.12]	1.94 [1.71-2.57]	**0.01**	1.99 [1.90-3.01]	2.23 [1.70-2.71]	1.86 [1.66-2.22]	**0.012**	0-2
T cells	-	0.99 [0.81-1.20]	1.26 [1.03-1.58]	**0.02**	1.35 [1.18-2.12]	1.31 [1.12-1.99]	1.19 [0.83-1.42]	**0.023**	0-1, 0-2
$CD4^+$	-	0.55 [0.48-0.74]	0.74 [0.64-1.04]	**0.001**	0.87 [0.58-1.33]	0.96 [0.64-1.24]	0.72 [0.64-0.81]	**0.001**	0-1, 0-2
$CD8^+$	-	0.30 [0.23-0.43]	0.44 [0.31-0.59]	**0.004**	0.43 [0.31-0.77]	0.43 [0.30-0.58]	0.46 [0.33-0.55]	**0.004**	0-3
B cells	-	0.28 [0.19-0.35]	0.14 [0.10-0.20]	**< 0.0001**	0.14 [0.09-0.15]	0.18 [0.10-0.24]	0.13 [0.08-0.18]	**< 0.0001**	0-1, 0-2, 0-3
NK cells	-	0.20 [0.15-0.33]	0.40 [0.30-0.58]	**< 0.0001**	0.38 [0.30-0.55]	0.37 [0.30-0.59]	0.42 [0.31-0.58]	**< 0.0001**	0-1, 0-2, 0-3
Neutrophils (G/L)	1,8-7,5	2.78 [2.47-3.46]	3.23 [2.33-3.90]	0.18	3.12 [2.36-4.65]	3.32 [2.44-4.15]	3.11 [2.29-3.68]	-	-
Eosinophils (G/L)	0,04-0,5	0.17 [0.11-0.22]	0.20 [0.12-0.28]	0.12	0.18 [0.10-0.26]	0.27 [0.16-0.41]	0.16 [0.12-0.25]	-	-
Basophils (G/L)	0-0,2	0.02 [0.02-0.03]	0.03 [0.01-0.04]	0.54	0.03 [0.02-0.05]	0.03 [0.02-0.04]	0.02 [0.01-0.03]	-	-
Monocytes (G/L)	0,2-1	0.43 [0.38-0.50]	0.59 [0.49-0.81]	**< 0.0001**	0.57 [0.55-0.82]	0.55 [0.38-0.72]	0.63 [0.50-0.82]	**< 0.0001**	0-1, 0-2, 0-3

Data are show as Mean ± SD or Median [interquartile range]. Statistical tests have been performed sequentially: (1) Mann & Whitney test (otherwise specified), (2) Kruskal-Wallis test (otherwise specified), if previous test significant, (3) Dunn post-hoc test, if previous test significant (0 = control group, 1,2,3 = groups exposed <5, 5-10, >10 years respectively). P-values have been adjusted according to Hochberg method to account for repeated analysis.

Supplemental Table 4. Regulatory T cells (Tregs), activated T cells and ratio of Tregs to activated cells, complete data.

		Not exposed to CS (n = 42)	All exposed subjects (n = 55)	Adjusted p-values (not exposed vs exposed)	Exposed < 5 years (n = 10)	Exposed 5-10 years (n = 18)	Exposed > 10 years (n = 27)	Adjusted p-values (4 groups comparisons)	Significant intergroup comparison
Tregs	%	6.81 [5.54-8.06]	5.55 [4.61-6.72]	**0.02**	6.03 [5.08-6.98]	5.88 [4.57-7.36]	5.38 [4.61-6.56]	**0.046**	0-3
	Count	44.56 [35.81-56.3]	42.70 [32.27-53.42]	0.72	44.05 [35.69-77.49]	47.81 [39.38-58.63]	34.41 [30.2-47.04]	-	-
$CD3^+HLA\text{-}DR^+$	%	7.19 [6.12-9.76]	9.39 [7.04-13.05]	0.09	7.2 [5.62-9.81]	10.1 [6.77-12.4]	11.3 [8.29-14.55]	-	-
	Count	72.28 [56.39-96.32]	128.41 [94.6-182.75]	**0.0002**	108.18 [80.38-177.88]	139.26 [107.81-182.65]	128.41 [94.54-204.04]	**0.002**	0-2, 0-3
$CD4^+HLA\text{-}DR^+$	%	5.32 [4.18-6.05]	6.46 [5.08-8.34]	**0.046**	6.24 [4.35-7.31]	6.55 [5.08-9.05]	6.46 [5.54-8.26]	0.057	-
	Count	28.03 [19.74-37.5]	51.36 [40-70.69]	**< 0.0001**	48.89 [39.11-82.53]	59.54 [51.36-71.77]	40.27 [37.06-67.81]	**< 0.0001**	0-1, 0-2, 0-3
$CD8^+HLA\text{-}DR^+$	%	11.8 [8.46-14.9]	14.0 [10.15-21.15]	0.16	9.5 [8.81-16]	15.95 [9.46-18.6]	14.1 [11.55-22.1]	-	-
	Count	36.72 [22.45-50.72]	59.24 [39.33-92.27]	**0.004**	54.92 [33.33-80.57]	52.32 [37.02-81.16]	65.62 [45.82-113.52]	**0.012**	0-3
Tregs / $CD3^+HLA\text{-}DR^+$ ratio	%	0.87 [0.67-1.1]	0.58 [0.41-0.78]	**0.004**	0.88 [0.78-1.13]	0.61 [0.45-0.75]	0.52 [0.37-0.64]	**0.002**	0-3, 1-3
	Count	0.54 [0.38-0.67]	0.35 [0.24-0.49]	**0.007**	0.54 [0.48-0.73]	0.38 [0.28-0.48]	0.28 [0.21-0.41]	**0.003**	0-3
Tregs / $CD4^+HLA\text{-}DR^+$ ratio	%	1.36 [0.89-1.5]	0.82 [0.71-1.05]	**0.001**	1.06 [0.96-1.41]	0.79 [0.67-0.84]	0.86 [0.70-1.00]	**0.002**	0-2, 0-3
	Count	1.36 [0.91-1.77]	0.83 [0.71-1.06]	**0.001**	1.08 [0.9-1.46]	0.76 [0.64-0.88]	0.83 [0.72-1.02]	**0.004**	0-2, 0-3
Tregs / $CD8^+HLA\text{-}DR^+$ ratio	%	0.56 [0.44-0.76]	0.39 [0.25-0.6]	0.052	0.56 [0.48-0.86]	0.41 [0.26-0.68]	0.34 [0.24-0.45]	-	-
	Count	1.07 [0.79-1.72]	0.73 [0.41-1.37]	0.09	1.42 [1.28-1.81]	0.91 [0.61-1.54]	0.56 [0.41-1.06]	-	-

Data are shown as Median [Interquartile range]. Count is in cells/µL. Statistical tests have been performed sequentially: (1) Mann & Whitney test, (2) Kruskal-Wallis test, if previous test significant, (3) Dunn post-hoc test, if previous test significant (0 = control group, 1,2,3 = groups exposed <5, 5-10, >10 years respectively). P-values have been adjusted according to Hochberg method to account for repeated analysis.
